# Imaging Diagnosis for Left Ventricular Thrombosis in Idiopathic Hypereosinophilic Syndrome: Two Case Reports

**DOI:** 10.1097/MD.0000000000000082

**Published:** 2014-09-26

**Authors:** Yu-Quan He, Ya-Nan Zhao, Jin-Ming Zhu, Meng-Chao Zhang, Lin Liu, Hong Zeng, Ping Yang

**Affiliations:** Division of Cardiology (Y-QH, Y-NZ, J-MZ, HZ, PY); and Division of Radiology (M-CZ, LL), China-Japan Union Hospital of Jilin University, Changchun, Jilin, China.

## Abstract

Idiopathic hypereosinophilic syndrome (IHES) is a rare disease that is frequently associated with cardiac thrombosis and endocardial wall thickness.

This case report describes 2 patients who had IHES associated with left ventricular (LV) thrombi. The patients’ symptoms are atypical. Peripheral blood and bone marrow tests showed markedly elevated eosinophils. Electrocardiography showed ischemic changes in both patients. Negative computed tomography (CT) angiography excluded coronary artery stenosis. Transthoracic echocardiography (TTE), conventional multislice spiral CT, gemstone spectral CT, and cardiac magnetic resonance imaging were used to identify the LV intraluminal thrombus and endocardial thickening, and the diagnostic values of each imaging method were analyzed and compared.

These patients were clinically diagnosed as “IHES, LV thrombosis, NYHA heart function classification I.”

Both patients received oral prednisone and warfarin therapy.

At 5 month follow-up, TTE rechecks showed that the size of the LV thrombotic lesion was reduced in the first case but substantially increased in the second case.

## INTRODUCTION

Idiopathic hypereosinophilic syndrome (IHES) is a rare clinical disease that has high morbidity and mortality due to its high tendency for hypercoagulation and associated thromboembolic complications in vital organs such as the heart, brain, lungs, and peripheral vessels.^1,2^ The heart is frequently involved in IHES.^3–5^ Chusid et al^6^ found that IHES was associated with cardiac thrombosis in up to 41% of patients. There are currently several case reports on IHES that are associated with peripheral and cardiac thromboembolism.^2,5^ Here, we examine a different viewpoint from previous studies, and analyze and compare the characteristic manifestations and features of left ventricular (LV) intraluminal thrombus in patients with IHES based on transthoracic echocardiography (TTE), conventional multislice spiral computed tomography (MSCT), gemstone spectral computed tomography (CT), and cardiac magnetic resonance imaging (MRI) images.

### Case 1

#### Patient Information

A 56-year-old man was admitted to our hospital on June 11, 2013 complaining of intermittent tightness in his chest, shortness of breath, and facial edema over the last 2 years, with acute exacerbation for 7 days. The patient had no history of smoking, drinking alcohol, or drug or food allergy. The patient did not receive any diagnosis and treatment before admission.

#### Clinical Findings

On admission, his body temperature was 36.5°C and blood pressure was 104/60 mm Hg. The patient appeared chronically ill with slight cyanosis of the lips and facial swelling with no distention of the jugular veins, and no dry or moist lung rales were heard. The cardiac boundary was normal, and the patient’s heart rate was 72 beats/min with a regular rhythm; no murmur was noted in the auscultatory valve areas. No liver and spleen enlargement or edema was noted in the lower extremities.

#### Diagnostic Focus and Assessment

**FIGURE 1:**
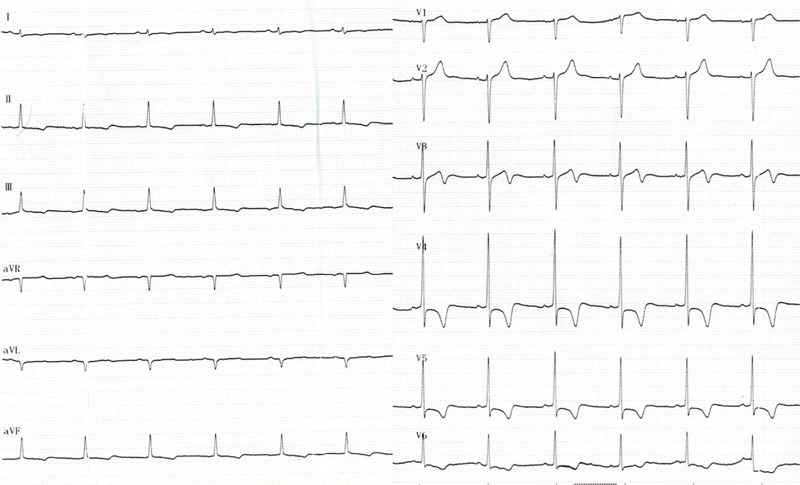
12 lead ECG recording of the first patient. Leads I, II, III, aVR, aVL, and aVF are shown on the left, and leads V_1_–V_6_ are shown on the right (from top to bottom). ECG = electrocardiography.

**FIGURE 2:**
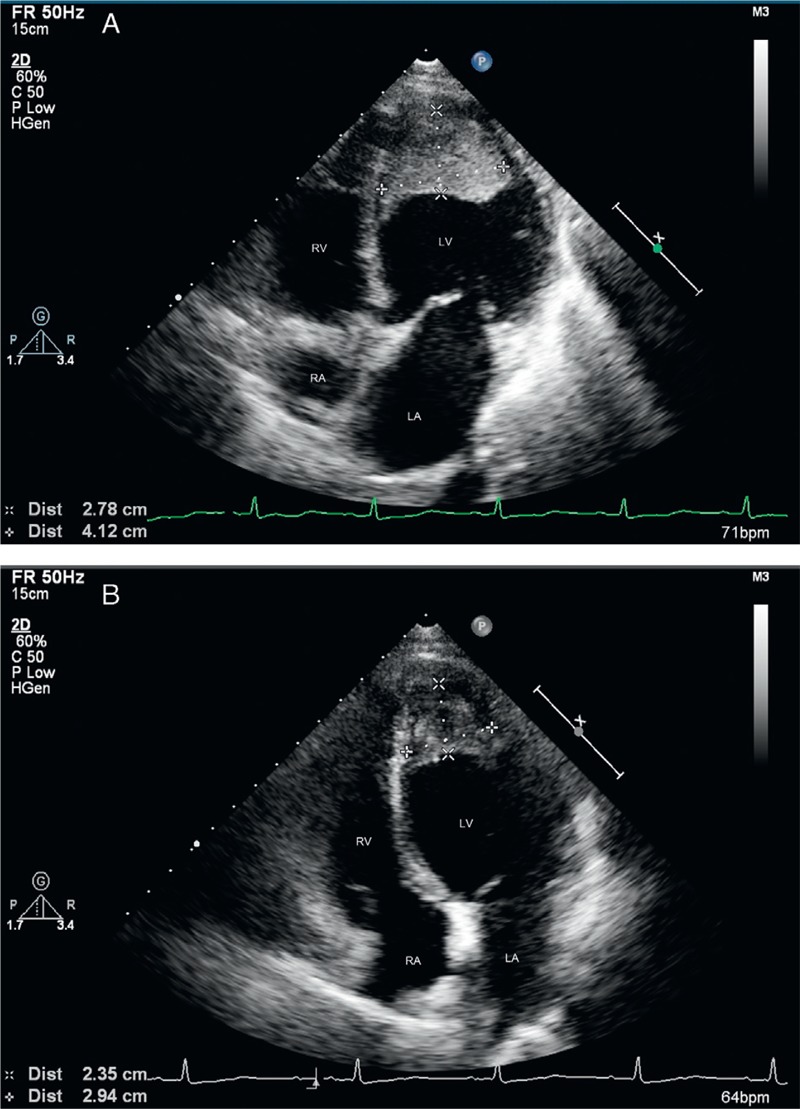
Images of 2D TTE examination of the first patient. (A) shows a half-moon-shaped heterogeneous echo of a thrombus measuring 41.2 × 27.8 mm at the LV apex. (B) shows that the size of the thrombus was decreased to 29.4 × 23.5 mm at 5 month follow-up. 2D = 2-dimensional, LV = left ventricular, TTE = transthoracic echocardiography.

**FIGURE 3:**
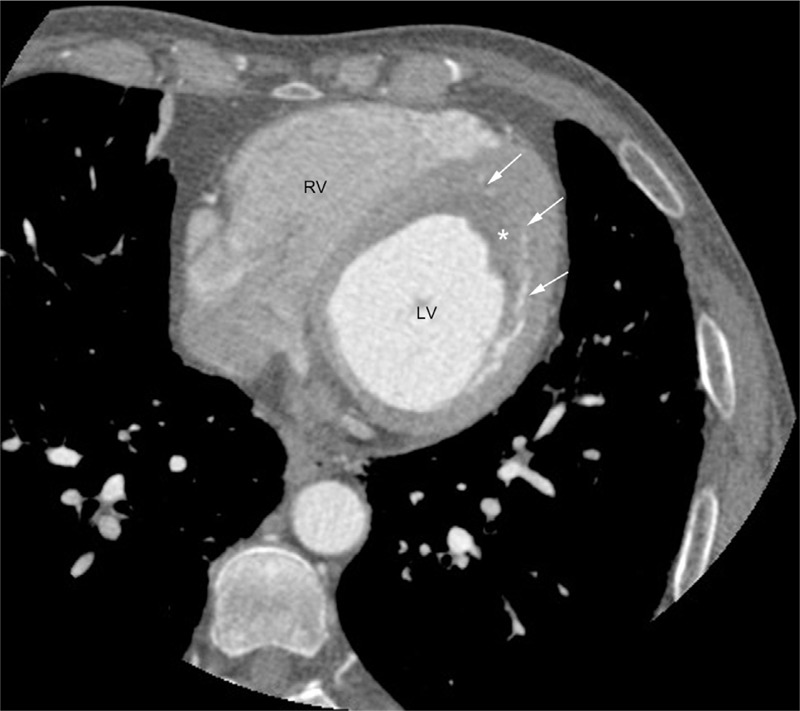
Images of conventional 64-row MSCT of the first patient. Image of the diastolic short axis shows a low-density shadow of filling defects at the LV apex (∗ delineates the contour of the thrombus; white arrows indicate the borders of the thrombus). LV = left ventricular, MSCT = multislice spiral computed tomography.

Routine blood test results showed a white blood cell count of 24.1 × 10^9^/L with 70.8% eosinophils at the absolute count of 17.1 × 10^9^/L. Bone marrow aspiration results showed 0.5% promyelocytes, 2.5% eosinophilic myelocytes, 10% eosinophilic metamyelocytes, 19% eosinophilic stab granulocytes, and 40% eosinophilic segmented granulocytes. No abnormality was found in urine, coagulation routine, liver and renal function, blood lipids, glucose, and electrolytes. The patient was negative for antibodies to hepatitis virus, *Treponema pallidum*, and human immunodeficiency virus (HIV). Tests for parasites, immunoglobulin, antinuclear antibody series, rheumatic factors, and complement revealed no abnormalities. Ultrasound examination of the liver, gallbladder, pancreas, spleen, and bilateral kidneys showed no abnormalities. Chest CT revealed interstitial pneumonia in bilateral lungs. Electrocardiography (ECG) showed ST-segment depression of approximately 0.1 mV in leads V_3_–V_6_ with inverted T waves in leads II, III, avF, and V_3_–V_6_ (Figure 1). TTE showed the normal cardiac chamber sizes and a heterogeneous echo from a half-moon shape solid mass that measured 41.2 × 27.8 mm and was identified at the LV apex; the mass was closely attached to the ventricular walls, especially the posterior interventricular septum (Figure 2A). The conclusive diagnosis from the TTE examination was “LV thrombosis; normal LV systolic and decreased diastolic function.” The patient underwent a contrast-enhanced 64-slice cardiac spiral CT scan (Discovery CT750 HD scanner; GE) with iopromide (Ultravist 370 mg/mL; Schering, Berlin, Germany) as the contrast agent. Three-dimensional (3D) reconstruction of the coronary arteries showed no abnormalities. At the LV apex, a low-density mass was observed, which had no clear boundary from its attachment to the LV endocardium (Figure 3). Thus, it was not feasible to correctly define the thickness of the endocardium that was involved.

**FIGURE 4:**
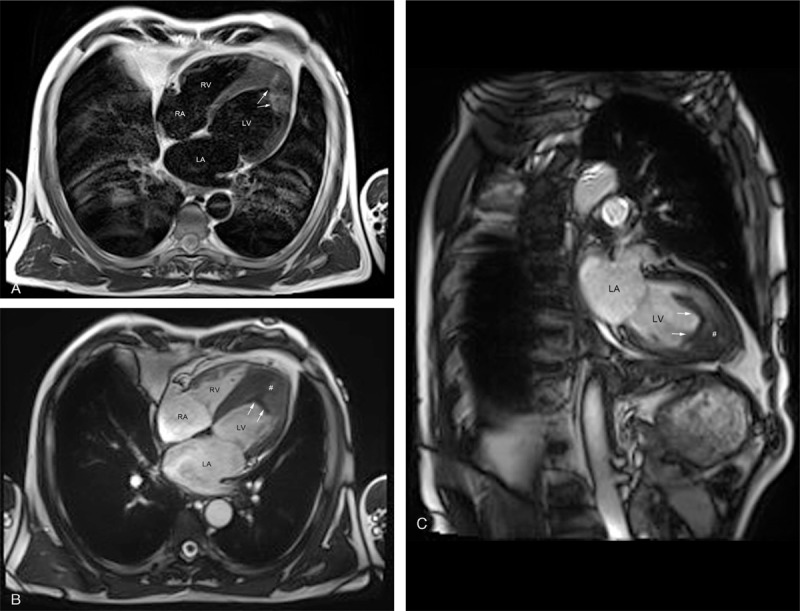
MRI images of black-blood TSE and bright-blood True FISP sequences of the first patient. (A) shows T_2_WI images with TSE sequence (white arrows indicate focal high-density signals; scan parameters, TR = 700 ms, TE = 30 ms, slice thickness = 5 mm, field of view = 340 × 340 mm, and flip angle = 180° for T_1_WI and TR = 800 ms, and TE = 77 ms for T_2_WI). (B) and (C) show single images of cine-MRI in the LV 4-chamber and 2-chamber long axis plane with the True FISP sequence, respectively (white arrows indicate the borders of the thrombus; # delineates the thickened endocardium; scan parameters, TR = 40 ms, TE = 1.4 ms, slice thickness = 6 mm, field of view = 340 × 340 mm, and flip angle = 57°). LV = left ventricular, MRI = magnetic resonance imaging, T_1_WI = T1-weighted imaging, T_2_WI = T2-weighted imaging, True FISP = true fast imaging with steady-state precession, TSE = turbo spin echo.

**FIGURE 5:**
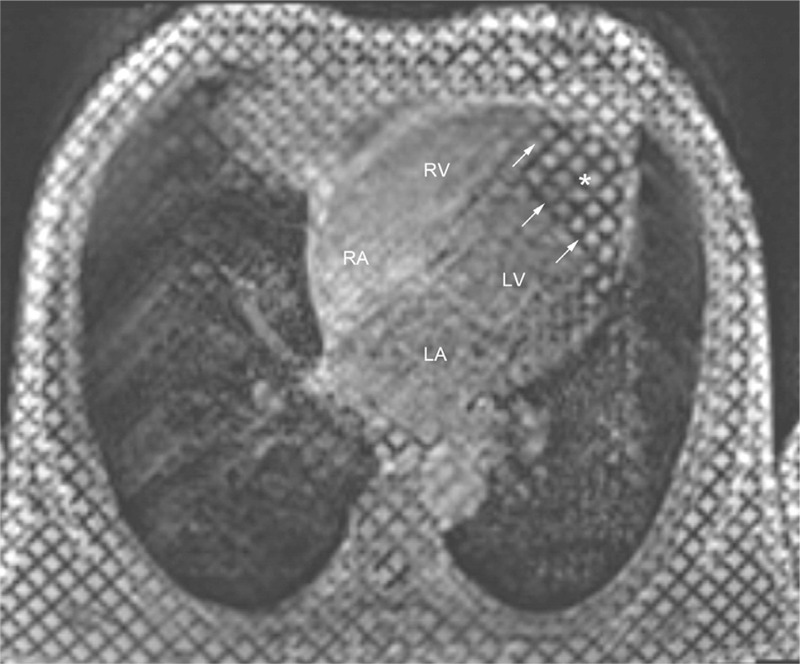
End-systolic tagging cine-MRI image in the LV 4-chamber long axis plane. Singe tagging image shows decreased myocardial systolic function in the LV apex area (lack of grid-deformation; ∗ delineates the contour of the thrombus; white arrows indicate the borders of the thrombus). LV = left ventricular, MRI = magnetic resonance imaging.

Cardiac cine-MRI was performed for this patient using a Skyra 3.0T MR scanner (Siemens, Erlangen, Germany) with breath-hold, retrospective ECG gating, and bright-blood true fast imaging with steady-state precession (True FISP) sequences. Tagging cine-MRI was performed using grid-tagged sequences, provided by the preinstalled “dot” program of Siemens (Erlangen, Germany). In addition, T1-weighted imaging (T_1_WI) and T2-weighted imaging (T_2_WI) were performed using black-blood turbo spin echo (TSE) sequence. Both T_1_WI and T_2_WI images clearly showed the focal endocardial thickening at the LV apex, and the T_2_WI image also revealed focal heterogeneous high-density signals in the myocardium at the LV apex, highly suggestive of local active inflammatory edema (Figure 4A). Single cine-MRI images showed a low-signal solid mass at the LV apex and a marked thickening of its attached endocardium (Figure 4B and C). Because MRI has a high tissue resolution, the MRI signals obtained for the normal LV cavity, LV intraluminal thrombus, and thrombus-attached endocardium were different, thereby, facilitating the identification and measurement of thrombus size and the endocardial thickness. The grid-tagged cine-MRI images revealed intramyocardial dysfunction, as evidenced by the lack of tag grid deformation in the areas of the LV thrombus and its attached myocardium (Figure 5).

#### Therapeutic Intervention and Follow-Up

The patient received oral prednisone and warfarin therapy, and a TTE recheck after 5 month showed that the LV thrombus lesion was markedly reduced (Figure 2B).

### Case 2

#### Patient Information

A 41-year-old man was admitted to hospital on December 27, 2013, complaining of intermittent chest pain and chest tightness for 1 year, with acute exacerbation and high fever for 12 days. The patient had a history of smoking for 28 years with no history of drug or food allergy. The patient did not see any doctors before admission.

#### Clinical Findings

On admission, his body temperature was 37.4°C and blood pressure was 100/60 mm Hg. The patient appeared acutely ill with no cyanosis of the lips or jugular venous distention. Few moist rales were heard at the base of the right lung. The patient’s heart rate was 74 beats/min with a regular rhythm, and a light holosystolic blowing murmur with 2/6 degree was heard at the auscultatory mitral area. No liver and spleen enlargement or edema of the lower limbs was found.

#### Diagnostic Focus and Assessment

**FIGURE 6:**
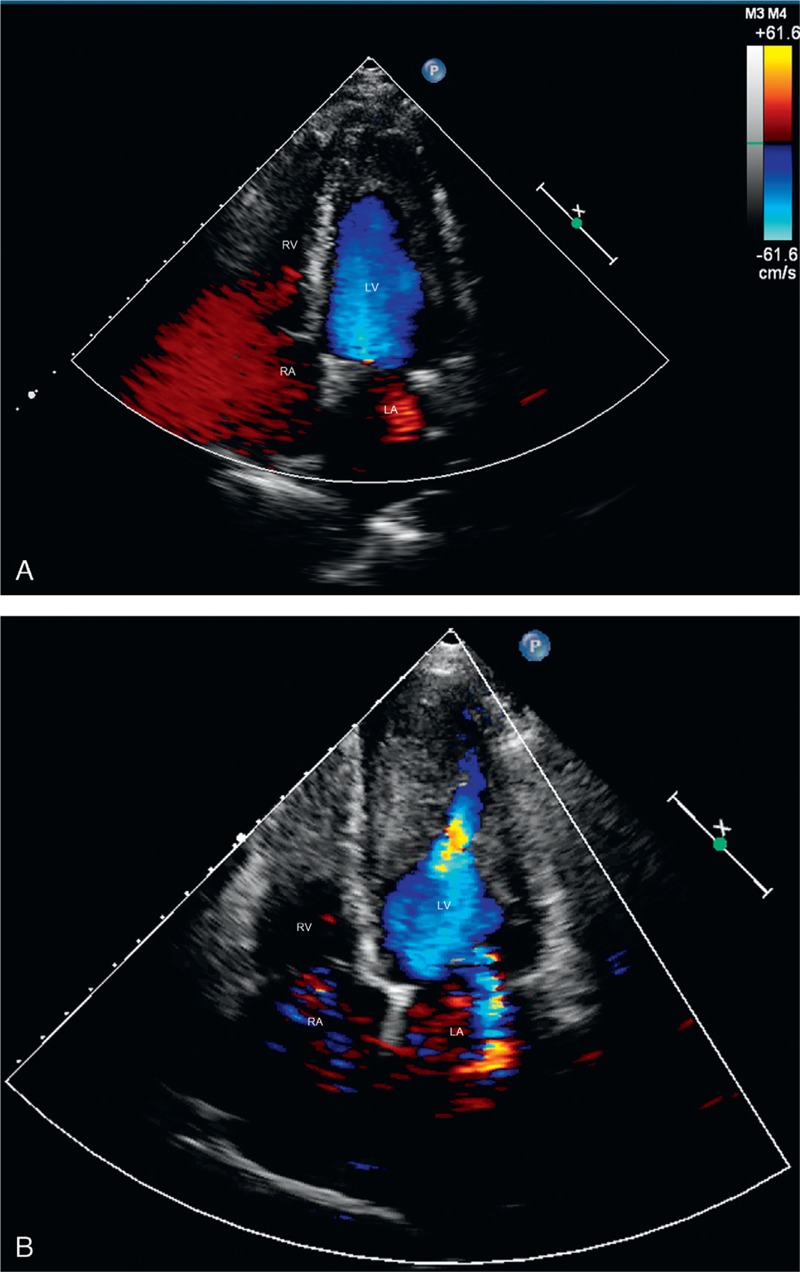
Images of 2D TTE examination of the second patient. Image (A) shows cap-shaped echoes of a mural thrombus at the apex and middle part of the LV cavity. Image (B) shows that at 5 month follow-up, the size of the thrombus was increased substantially whereas blood flow could be found in the central thrombus during the diastole phase. 2D = 2-dimensional, LV = left ventricular, TTE = transthoracic echocardiography.

Routine blood test results showed a white blood cell count of 50.66 × 10^9^/L, with 71.5% eosinophils at the absolute count of 36.22 × 10^9^/L. Bone marrow biopsy showed 0.5% neutrophilic myelocytes, 0.5% eosinophilic myelocytes, 12.5% eosinophilic metamyelocytes, 15.5% eosinophilic stab granulocytes, and 49% eosinophilic segmented granulocytes. Other results included troponin-I, 3.17 ng/mL; aspartate aminotransferase, 76 IU/L; lactate dehydrogenase, 1173 IU/L; blood urea nitrogen, 10.6 mmol/L; dimerized plasmin fragment D, 845.0 ng/mL; C-reactive protein, 18.3 mg/L; and brain natriuretic peptide, 6120.00 pg/mL. No abnormalities were found in coagulation function, serum creatinine, electrolytes, blood lipids and glucose, and routine urine. Results for the hepatitis virus, *T pallidum*, and HIV antibodies were negative. No abnormalities in parasites, thyroid function, rheumatic factors, and immunological indexes were found. Ultrasound examination of the liver, spleen, pancreas, bilateral kidneys, and adrenal gland showed no abnormalities. Chest CT revealed a small amount of right pleural effusion. ECG illustrated ST-segment depression >0.3 mV in leads V_1_–V _6_ with deep inverted T waves (figure not shown). TTE showed normal chamber sizes, and a cap-shaped echo of mural thrombus that measured 37.5 × 27.1 mm was observed at the apex and middle part of the LV cavity; it was closely attached to the ventricular wall (Figure 6A). Mitral valve regurgitation was noted with an area of 2.5 cm^2^. The conclusive diagnosis of the TEE exam was “LV mural thrombosis, mild mitral regurgitation, normal LV systolic and diastolic functions.”

**FIGURE 7:**
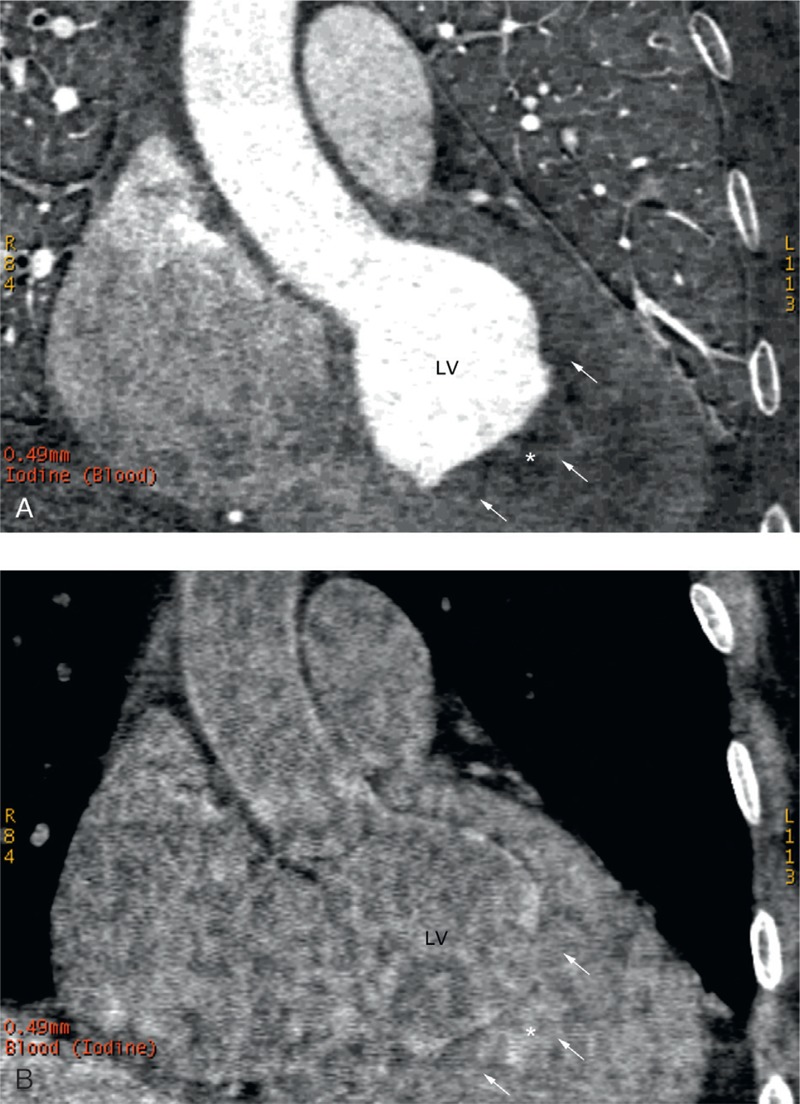
Contrast enhanced iodine- and blood-based substance separation images of gemstone spectral CT of the second patient. (A) and (B) show iodine- and blood-based density images, respectively (∗ delineates the contour of the thrombus; white arrows indicate the borders of the thrombus). CT = computed tomography, LV = left ventricular.

A contrast-enhanced spectral CT scan was performed using the newly developed gemstone spectral imaging (GSI) mode of the Discovery CT750 HD CT scanner (GE, Milwaukee, WI) with Iopromide (Ultravist 370 mg/mL; Schering) as the contrast agent. The scanning parameters were as follows: helical scanning speed of the x-ray tube, 0.6 s/r; pitch, 0.969; detector width, 4.0 cm; instantaneous voltage switch (0.5 ms) between high and low energy (140 and 80 kVp); and tube current, 600 mA. In the GSI mode, iodine and blood were chosen as the base material pair. In the images of iodine-based density, the normal LV cavity filled with the contrast agent was displayed as high-brightness signals whereas the LV thrombus was manifest as filling defects. In the images of blood-based density, the LV thrombus signal intensity was similar to that of normal LV cavity filled with blood, both displaying relatively high-brightness gray shadows (Figure 7).

**FIGURE 8:**
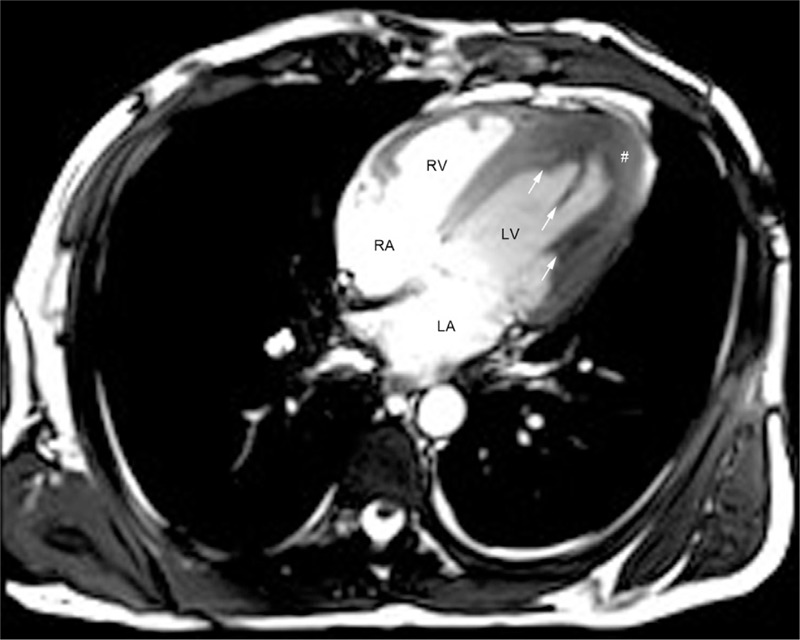
Single image of cine-MRI with True FISP sequence in the LV 4-chamber long axis plane of the second patient (white arrows indicate the borders of the thrombus; # delineates the thickened endocardium). LV = left ventricular, MRI = magnetic resonance imaging, True FISP = true fast imaging with steady-state precession.

Cardiac cine-MRI was performed using the same instrument and methods with similar parameter settings as in case 1. A single cine-MRI image clearly showed a marked thickening of the LV wall and annular low-density shadows at the apex, septum, and lower lateral wall of the LV, with a linear low-density shadow extending into the center of the LV cavity (Figure 8). In a detailed analysis of the image, we found that the obtained MRI signals were different among the normal LV cavity, LV intraluminal thrombus, and thrombus-attached endocardium. Thus, compared with TTE and CT scan, cardiac MRI is the most favorable to define the thrombus size and assessment of the endocardial thickness.

#### Therapeutic Intervention and Follow-Up

The patient was prescribed with warfarin and prednisone, but TTE recheck at 5 month follow-up showed that the size of the thrombus was increased substantially, whereas blood signals could be found flowing into the central thrombus probably because of the potent anticoagulation activity of warfarin (Figure 6B).

## DISCUSSION

IHES, a rare disease in cardiovascular medicine, is characterized by persistent and unexplained peripheral eosinophilia of >1.5 × ^9^/L for >6 months with evidence of organ damage.^7^ It received much attention from clinicians because it is associated with severe thromboembolic complications in vital organs and relatively high mortality and morbidity.^1,2^ The heart is one of the organs most frequently involved in IHES. Heart involvement occurs in 3 stages: the early necrotic stage, the intermediate thrombotic stage, and the final fibrotic stage.^2,4^ IHES may ultimately progress to restrictive cardiomyopathy and valvular insufficiency because of fibrosis and scar formation in the myocardium and endocardium. Here, we report 2 cases of typical LV intraluminal thrombosis associated with IHES. From a viewpoint of cardiac diagnostic imaging, we analyzed and compared the unique diagnostic value and advantages of TTE, conventional MSCT, gemstone spectral CT, and cardiac MRI for the detection of LV intraluminal thrombus in IHES.

Currently, TTE examination is the most commonly used, economical, and simple means to clinically diagnose cardiovascular diseases. Typical TTE findings of heart involvement associated with IHES are endocardial thickening, cardiac mural thrombus, and restrictive diastolic (filling) dysfunction because of the thickened endocardium.^3,4,8^ In the present study, TTE first identified LV intraluminal thrombus in both patients. In case 1, the mitral Doppler flow velocity measurement indicated LV diastolic dysfunction. In case 2, concomitant mitral regurgitation was noted, suggesting that the IHES lesion might have attacked chordae tendineae or the papillary muscle. The above positive results of TTE provided valuable information for identification and assessment of heart damage associated with IHES. However, because of the low tissue resolution, it is not feasible to correctly identify the interface between a thrombotic lesion and its attached thickened endocardium. Additionally, there is a large amount of variation among operators who perform TTE examinations. Other studies have shown that up to 46% of TTE examinations have an uncertain diagnosis of thrombosis.^9^ Despite this limitation, TTE provides a cost-effective, simple, safe, noninvasive, and nonradioactive method, which is valuable for the preliminary screening of thrombi and for observing the curative effect of thrombolytic therapies during follow-up.

In this article, both patients have symptoms such as shortness of breath or chest pain. ECG showed characteristic ischemic changes, and myocardial injury markers elevated in the second patient. Both patients were initially suspected of having “acute coronary syndrome,” but the negative CT angiogram excluded coronary artery stenosis. Although clearly showing the 3D coronary arteries, cardiac CT imaging is also capable of showing the low-density shadows or filling defects caused by thrombotic lesions.^10^ In case 1, a conventional nonspectral MSCT scan clearly visualized the thrombus-induced filling defects in terms of morphology, location, and size, but this approach could not either make a qualitative diagnosis based on the thrombus tissue composition, or accurately differentiate the thrombotic lesion from normal papillary muscles, which was also manifest as a low-density shadow on routine MSCT images. In case 2, the newly developed gemstone spectral CT was used with iodine and blood as the chosen base material pair.^11,12^ Single material images of iodine- and blood-based density were generated, based on the unique dual material spectral CT separation technique.^13,14^ In the isolated iodine-based density image, the LV thrombus presented as low-density shadows or filling defects, because of a lack of blood supply and thus the contrast agent (iodine) hardly reached the lesion central area of the thrombus. On the other hand, in the isolated blood-based density image, the LV thrombus and normal LV cavity presented with similar high-brightness signal intensities, probably due to the similar composition of thrombus and blood in the LV cavity (both contain blood components such as platelets and erythrocytes). Our recent study (H.Z. et al, Application of spectral CT dual-substance separation technology for diagnosing left ventricular thrombus. May 2014.) also found that the dual material spectral CT separation technique described above is useful for differentiating between the normal LV papillary muscles and thrombotic lesions. Our study showed that the normal papillary muscles presented as a similar low-density shadow or filling defect as the thrombotic lesion in the iodine-based density images, due to a lack of blood supply. However, because its composition is different from blood and the thrombus, papillary muscle presents as a low-density shadow in blood-based density images, whereas both the LV cavity and thrombotic lesion showed similar high-brightness gray shadows. Our limited case report presented in this article may not be sufficient for discussing spectral CT utility; of course, these findings need further validation through clinical practice.

To further define the nature, location, extent, and severity of the heart involvement and obtain information such as cardiac function and myocardial motion, both patients underwent cardiac cine-MRI scans using the bright-blood True FISP sequence. In both cases, single-slice cine images demonstrated extremely high tissue resolution, evidenced by the different MRI signals obtained from the LV thrombus, normal LV cavity, and thrombus-attached endocardium, thereby facilitating the correct measurement of thrombus size and assessment of attached endocardial thickness.

In clinical practice, an intracardiac thrombus can be removed by surgical resection.^15^ However, a distinct plane between the thrombus and its adjacent endocardium should exist for the thrombus to be removed. Due to MRI’s high tissue resolution, it is of great value for preoperative therapeutic decision-making and selection of operative strategies.^16^ Cardiac MRI examination has another distinct advantage because it uses bright-blood sequences for cine imaging that do not require injected contrast agent. Additionally, we found that TSE T_2_WI imaging is sensitive for the detection of inflammatory edema, and the grid-tagged cine-MRI is beneficial for the assessment of myocardial motion dysfunctions.

For IHES patients who have heart involvement, correct evaluation of the scope and extent of myocardial necrosis and fibrosis is of great importance for disease staging, guiding patient treatment and estimating prognosis.^3,4^ Although images using edema-specific TSE T_2_WI sequences provide information on active myocardial edema, the existing imaging results in this article did not reveal sufficient information for myocardial necrosis and fibrosis. Iodine-enhanced delayed myocardial perfusion spectral CT imaging may be helpful.^10^ However, both our patients required 3D CT angiography; this scan was obtained in a few seconds after the concentration of the contrast agent (iodine) reached a peak level at the aortic root, and double cardiac CT scans exposed the patients to excessive radiation. Thus, this approach was not used for patients. In addition, delayed gadolinium-enhanced myocardial perfusion MRI is of great value for identifying and evaluating thrombi, necrosis, and fibrotic scars.^17,18^ Although these procedures were not performed for these patients, they are worthy of further study in the future.

Glucocorticoids and warfarin are the first-line drugs for IHES associated with cardiac thrombosis.^19^ At 5 month follow-up, the size of LV thrombus was reduced in the first case, but substantially increased in the second case. Cytotoxic agents such as hydroxyurea and α-interferon may be needed for these patients, and surgical thrombectomy and endocardial resection may be the options.^19^

In summary, although endomyocardial biopsy is the gold standard for diagnosis of cardiac attacks associated with IHES,^10^ our study demonstrated that timely, accurate, and valuable diagnosis of cardiac thrombosis associated with IHES can be made based on imaging tools such as TTE, gemstone spectral CT, and MRI, in combination with clinical manifestations and laboratory test results. TTE is not capable of detecting coronary artery stenosis, and because of its low tissue resolution, it cannot provide sufficient and comprehensive information for associated cardiac lesions with IHES. Cardiac MSCT has a unique advantage for 3D coronary angiography, and the newly developed gemstone spectral CT may play a critical role in qualitative and differential diagnosis of cardiac thrombus components. Compared with other methods, cardiac MRI with its high tissue resolution is valuable for lesion assessment and therapeutic guidance. Finally, we conclude that for IHES patients with suspected cardiac involvement, a combination of imaging methods such as TTE, spectral CT, and MRI provides accurate, comprehensive, and valuable information for disease diagnosis, treatment guidance, and prognostic evaluation, which requires further clinical research, application, and promotion.

### Ethical Review and Consent

Ethical approval was obtained from the ethics committee of the China-Japan Union Hospital of Jilin University. Written informed consent was obtained from both the patients for publication of this case report and any accompanying images.

## References

[1] Todd S Hemmaway C Nagy Z Catastrophic thrombosis in idiopathic hypereosinophilic syndrome. Br J Haematol. 2014;165:25. 10.1111/bjh.12729 24456103

[2] Gurgun A Tuluce K Tuluce SY Hypereosinophilic syndrome presenting with large left ventricular apical thrombus and pulmonary embolism. Echocardiography. 2011;28:E180–E182. 21854430 10.1111/j.1540-8175.2011.01479.x

[3] Lorenzoni R Cortigiani L Melosi A Cardiac involvement in idiopathic hypereosinophilic syndrome. Heart. 2002;87:5534. 10.1136/heart.87.6.553 PMC1767133 12010938

[4] Kleinfeldt T Nienaber CA Kische S Cardiac manifestation of the hypereosinophilic syndrome: new insights. Clin Res Cardiol. 2010;99:419–427. 20333409 10.1007/s00392-010-0144-8

[5] Buyuktas D Eskazan AE Borekci S Hypereosinophilic syndrome associated with simultaneous intracardiac thrombi, cerebral thromboembolism and pulmonary embolism. Intern Med. 2012;51:309–313. 22293809 10.2169/internalmedicine.51.6156

[6] Chusid MJ Dale DC West BC The hypereosinophilic syndrome: analysis of fourteen cases with review of the literature. Medicine (Baltimore). 1975;54:1–27. 1090795

[7] Simon HU Rothenberg ME Bochner BS Refining the definition of hypereosinophilic syndrome. J Allergy Clin Immunol. 2010;126:45–49. 20639008 10.1016/j.jaci.2010.03.042 PMC3400024

[8] Weller PF Bubley GJ The idiopathic hypereosinophilic syndrome. Blood. 1994;83:2759–2779. 8180373

[9] Srichai MB Junor C Rodriguez LL Clinical imaging, and pathologic characteristics of left ventricular thrombus: a comparison of contrast enhanced magnetic resonance imaging, transthoracic echocardiography and transesophageal echocardiography with surgical or pathological validation. Am Heart J. 2006;152:75–84. 16824834 10.1016/j.ahj.2005.08.021

[10] Chen CH Tsai IC Jan SL MDCT evaluation of cardiac involvement in hypereosinophilic syndrome: differentiating mural thrombus, infarcted, and noninfarcted myocardium by delayed-phase scanning. Tex Heart Inst J. 2011;38:166–169. 21494529 PMC3066800

[11] Matsumoto K Jinzaki M Tanami Y Virtual monochromatic spectral imaging with fast kilovoltage switching: improved image quality as compared with that obtained with conventional 120-kVp CT. Radiology. 2011;259:257–262. 21330561 10.1148/radiol.11100978

[12] Pessis E Campagna R Sverzut JM Virtual monochromatic spectral imaging with fast kilovoltage switching: reduction of metal artifacts at CT. Radiographics. 2013;33:573–583. 23479714 10.1148/rg.332125124

[13] Lv P Zhang Y Liu J Material decomposition images generated from spectral CT: detectability of urinary calculi and influencing factors. Acad Radiol. 2014;21:79–85. 24331268 10.1016/j.acra.2013.09.023

[14] Zhang XF Lu Q Wu LM Quantitative iodine-based material decomposition images with spectral CT imaging for differentiating prostatic carcinoma from benign prostatic hyperplasia. Acad Radiol. 2013;20:947–956. 23830601 10.1016/j.acra.2013.02.011

[15] Tefferi A Gotlib J Pardanani A Hypereosinophilic syndrome and clonal eosinophilia: point-of-care diagnostic algorithm and treatment update. Mayo Clin Proc. 2010;85:158–164. 20053713 10.4065/mcp.2009.0503 PMC2813824

[16] Bishop GG Bergin JD Kramer CM Hypereosinophilic syndrome and restrictive cardiomyopathy due to apical thrombi. Circulation. 2001;104:E3–E4. 11447094 10.1161/01.cir.104.2.e3

[17] Weinsaft JW Kim HW Shah DJ Detection of left ventricular thrombus by delayed-enhancement cardiovascular magnetic resonance. J Am Coll Cardiol. 2008;52:148–157. 18598895 10.1016/j.jacc.2008.03.041

[18] Porto AG McAlindon E Hamilton M Diagnosing cardiac involvement in the hypereosinophilic syndrome by cardiac magnetic resonance. Am J Cardiol. 2013;112:135–136. 23570610 10.1016/j.amjcard.2013.02.064

[19] Klion AD Bochner BS Gleich GJ Approaches to the treatment of hypereosinophilic syndromes: a workshop summary report. J Allergy Clin Immunol. 2006;117:1292–1302. 16750989 10.1016/j.jaci.2006.02.042

